# Abuse, dependence and withdrawal associated with fentanyl and the role of its (designated) route of administration: an analysis of spontaneous reports from Europe

**DOI:** 10.1007/s00228-022-03431-x

**Published:** 2022-12-16

**Authors:** Kathrin Jobski, Carsten Bantel, Falk Hoffmann

**Affiliations:** 1 Department of Health Services Research, Carl von Ossietzky University Oldenburg, Ammerländer Heerstr. 114-118, 26129 Oldenburg, Germany; 2 University Department of Anesthesiology, Critical Care, Emergency and Pain Medicine, Klinikum Oldenburg, Oldenburg, Germany

**Keywords:** Fentanyl, Pharmacovigilance, Abuse, Dependence, Withdrawal, EudraVigilance

## Abstract

**Purpose:**

Fentanyl, a highly potent synthetic opioid used in cancer and non-cancer pain, is approved for various routes of administration. In Europe, fentanyl consumption increased substantially in the last decades but information on abuse, dependence and withdrawal associated with fentanyl is scarce, especially with respect to its different formulations.

**Methods:**

We analysed case characteristics of spontaneous reports of suspected fentanyl-associated abuse, dependence or withdrawal from European countries recorded in the EudraVigilance database up to 2018 with respect to the (designated) routes of administration and potential indications.

**Results:**

A total of 985 reports were included (mainly from France and Germany) with 43% of cases referring to transdermal fentanyl. Median age was 45 years (48.8% female) and 21.6% had musculoskeletal disorders. Only 12.6% of those using transdermal fentanyl had a cancer diagnosis compared to 40.2% and 26.8% of those using intranasal and oral transmucosal fentanyl, respectively. Depression was common (10.7%) and highest in cases with musculoskeletal disorders (24.9%) as was the use of benzodiazepines. Overall, 39.5% of reports resulted in a prolonged hospital stay and for 23.2% a fatal outcome was recorded. The respective proportions were especially high in cases with musculoskeletal disorders (56.3% with prolonged hospitalisation) and in those using transdermal fentanyl (35.2% fatalities).

**Conclusions:**

In suspected cases of abuse, dependence or withdrawal, fentanyl was mainly used for non-cancer pain indications and most often as transdermal formulations. Depression and prolonged hospitalisations were common, especially in patients with musculoskeletal disorders, indicating a vulnerable patient group and complex treatment situations.

**Supplementary Information:**

The online version contains supplementary material available at 10.1007/s00228-022-03431-x.

## Introduction

Fentanyl is a synthetic opioid which is about 100-fold more potent than morphine [1]. Originally developed as a parenteral analgesic, it was introduced into clinical practice in 1963 [1, 2]. Since the 1990s, fentanyl has been used as a transdermal patch, and in 1998, the first commercially successful transmucosal immediate-release fentanyl (TIRF) preparation, a soluble lozenge, was introduced, later followed by buccal tablets, films and nasal sprays [2]. Transdermal fentanyl is indicated to treat severe persistent pain that requires continuous treatment, whether or not the pain is caused by cancer [3]. In contrast, TIRF products are only approved for the treatment of breakthrough pain in opioid-tolerant cancer patients [2], although several reports indicate their frequent use in non-cancer pain [4–6].

Overall, these various approved types of formulations and routes of administration make fentanyl a unique opioid. However, the same properties that makes these medications effective and useful analgesics also impart to them desirability for abuse [7]. In the USA, fentanyl and its (licit and illicit) derivates have been considered recent drivers of the opioid epidemic with significant increases in overdose deaths [8, 9]. In most parts of Europe, fentanyl is the most frequently used step 3 opioid (according to the World Health Organisation’s analgesic ladder [10]), and increased fentanyl consumption has largely contributed to the rises in total opioid consumption, particularly since the early 2000s [11].

However, two recent studies observed that, in contrast to the USA, fentanyl does not seem to be a major driver of opioid use disorder across Europe [8] and that fentanyl and its derivatives, either diverted from medical use or illicitly manufactured, play a relatively minor role in deaths and acute poisoning [12]. The authors of the latter study, however, conclude that instruments of the current European drug monitoring system (e.g. drug-related emergency visits and deaths or monitoring of entrants to specialist drug treatment centres) may not be sufficiently sensitive for detecting important trends in the misuse of medicinal products [12]. Besides missing information on potential misuse/opioid use disorder in fentanyl users, insight on dependence or withdrawal associated with fentanyl is scarce. It is further unknown, whether the various fentanyl formulations play a role in this context. Here, spontaneous reports from all over Europe with in-depth information on the (mis)used products spanning many years may close a gap. Although a recent study assessed fentanyl misuse reported to adverse reporting systems from Europe and the USA [13], no studies examined problematic fentanyl use/misuse with respect to its different formulations.

Therefore, the aim of this present study was to analyse spontaneous reports of suspected fentanyl abuse, dependence or withdrawal in Europe focusing on the different routes of administration.

## Methods

### Study design and data source

We performed a retrospective analysis based on reports of suspected abuse, dependence or withdrawal associated with fentanyl. Data source was the EudraVigilance database which collects information on suspected adverse reactions for authorised medicines in the European Economic Area (EEA) [14]. The database has a core role in analysing safety profiles of medicinal products once they are used in everyday medical practice, and scientific publications based on EudraVigilance data have increased since 2010 [14]. The database has been used previously to examine abuse and dependence, e.g. with respect to fentanyl [13] or Z-drugs [15].

Following a formal request, we received a subset of Individual Case Safety Report (ICSR) data elements (cases) coded using the Medical Dictionary for Regulatory Activities (MedDRA) terminology [16].

### Case identification and included variables

Cases were based on the narrow search of the Standardised MedDRA Query (SMQ) “drug abuse, dependence and withdrawal” (preferred terms are displayed in Supplementary Table 1). SMQs are groupings of MedDRA terms that relate to a defined medical condition/area of interest and are intended to aid in the identification and retrieval of potentially relevant ICSR [16]. A narrow search approach yields cases that are highly likely to represent the condition of interest. We included all reports referring to fentanyl as a suspected or interacting drug allowing the free base (e.g. in matrix patches and other transdermal systems) as well as fentanyl’s salt forms such as citrate (the active ingredient of products administered intravenously or via the oral or nasal mucosa).

The product’s designated route of application (i.e. transdermal, oral transmucosal, intranasal or intravenous) was assessed from several variables including the pharmaceutical form (e.g. patch, lozenge, nasal spray, injection) as well as the product’s brand name (e.g. Durogesic^®^, Instanyl^®^). The EudraVigilance variable “route of administration” was used if its combination with the corresponding dosage led to an unambiguous designated route. Additionally, a reported transdermal route of administration was classified as such, since, e.g. a respective (mis)use of a fentanyl nasal spray was considered unlikely. Lastly, we examined whether free text information referred to specific routes (e.g. “misused a fentanyl patch”). Reports based on products referring to different designated routes of administration were classified as “multiple routes”. In all other cases, the designated route of application was considered “unknown”. For a designated transdermal route of administration, we additionally examined if there was evidence for another actual route based on different free text variables and including terms used in a study examining drug tampering for nonmedical opioid consumption [17].

We retrieved demographic information including age, sex and the country of the event’s occurrence from the respective variables. The start of the adverse reaction was used to determine its date (year). If this date was not available, the date when the report was first received by Eudravigilance was used instead. Furthermore, the reporter’s qualification was assessed in a hierarchical manner (physician, pharmacist, other health professional, consumer or other non-health professional).

Comorbidity and (potential) indication were assessed from the respective variables. The latter were restricted to the system organ classes (SOC) “neoplasms benign, malignant and unspecified (including cysts and polyps)” and “musculoskeletal and connective tissue disorders”. Furthermore, we examined whether cases had a history of “drug abuse, dependence or withdrawal”, “depression (excluding suicide and self-injury)” or “suicide, self-injury” based on the terms referring to the respective SMQs. Assigning anatomical therapeutic chemical codes (ATC) to each drug, we included the following comedication: antidepressants (ATC: N06A), antipsychotics (N05A), benzodiazepines (N03AE, N05BA, N05CD, N05CF), other opioids (N02A excluding fentanyl) and drugs for opioid dependence (N07BC). We further assessed whether the use of recreational drugs such as alcohol, cannabis or cocaine was reported. Lastly, for fatal cases, we examined causes of death also using the data fields referring to autopsy results.

### Statistical analyses

Firstly, we analysed the number of reports of suspected abuse, dependence or withdrawal associated with fentanyl use by route of administration, year and reporting country. We used descriptive statistics (median, interquartile range (IQR) and percentages), to display case and treatment characteristics by potential indication (musculoskeletal/connective tissue disorders vs. neoplasms), route and outcome (fatal vs. non-fatal). We separately compared fatal and non-fatal cases of users of transdermal fentanyl focusing on the patches’ strengths and on the actual route of administration.

Patients with missing information were excluded from the respective analyses resulting in different denominators.

All statistical analyses were performed using SAS, Version 9.4 (SAS Institute Inc).

## Results

### Overall characteristics

**Fig. 1:**
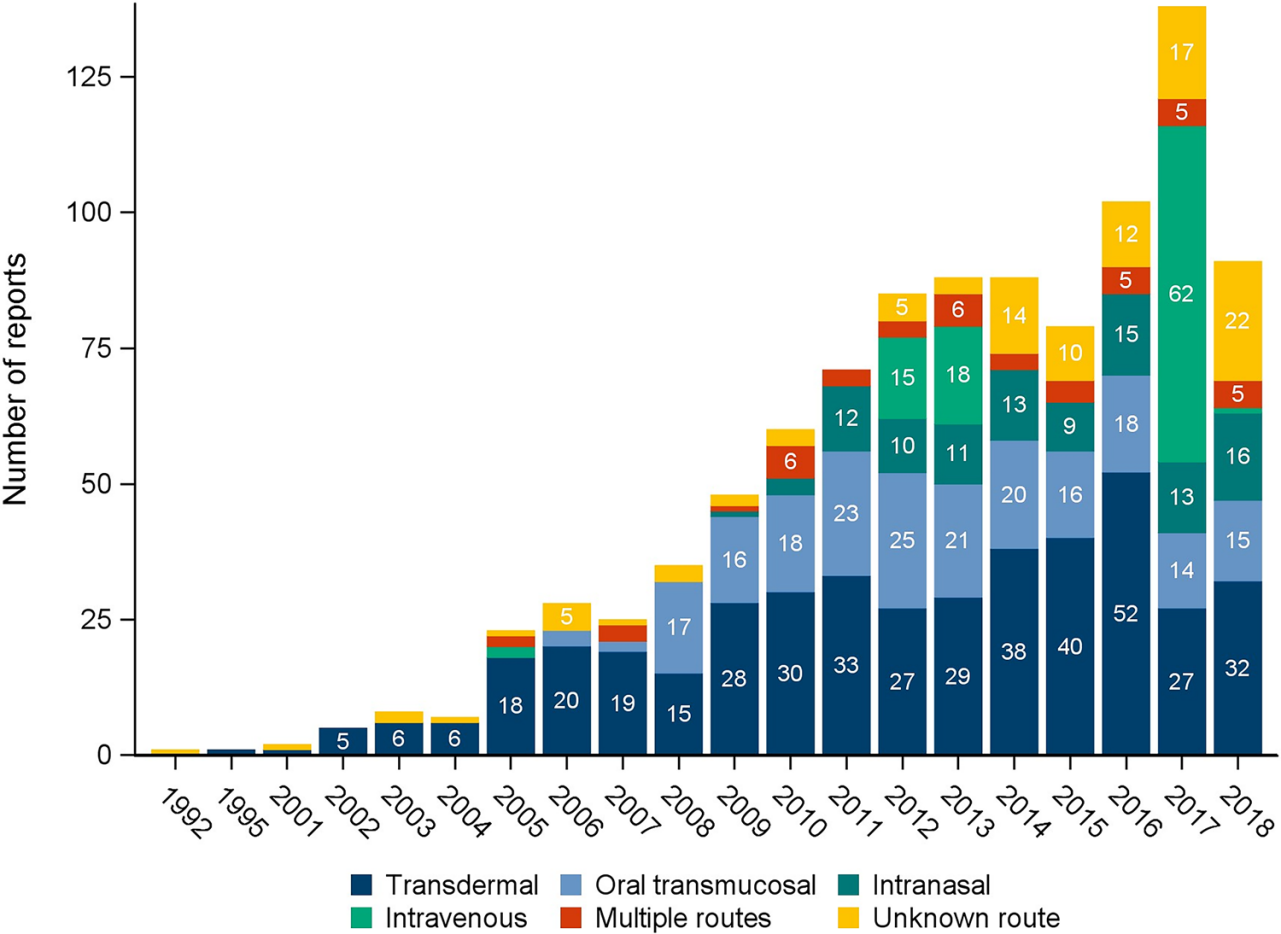
Reports of suspected fentanyl-associated abuse, dependence or withdrawal in Europe by year and (designated) route of administration (N = 985)

Eudra Vigilance received 985 European reports of suspected fentanyl abuse, dependence or withdrawal (1992–2018) with a maximum of 138 reports in 2017 (Fig. 1). About 43% of cases referred to transdermal fentanyl, whereas products administered via the oral or nasal mucosa were reported less often (21.2% and 10.5%, respectively). Intravenous fentanyl was stated in nearly 10% of cases, 4.7% referred to multiple routes and for 10.5% of cases no (designated) route of administration could be determined.

**Fig. 2:**
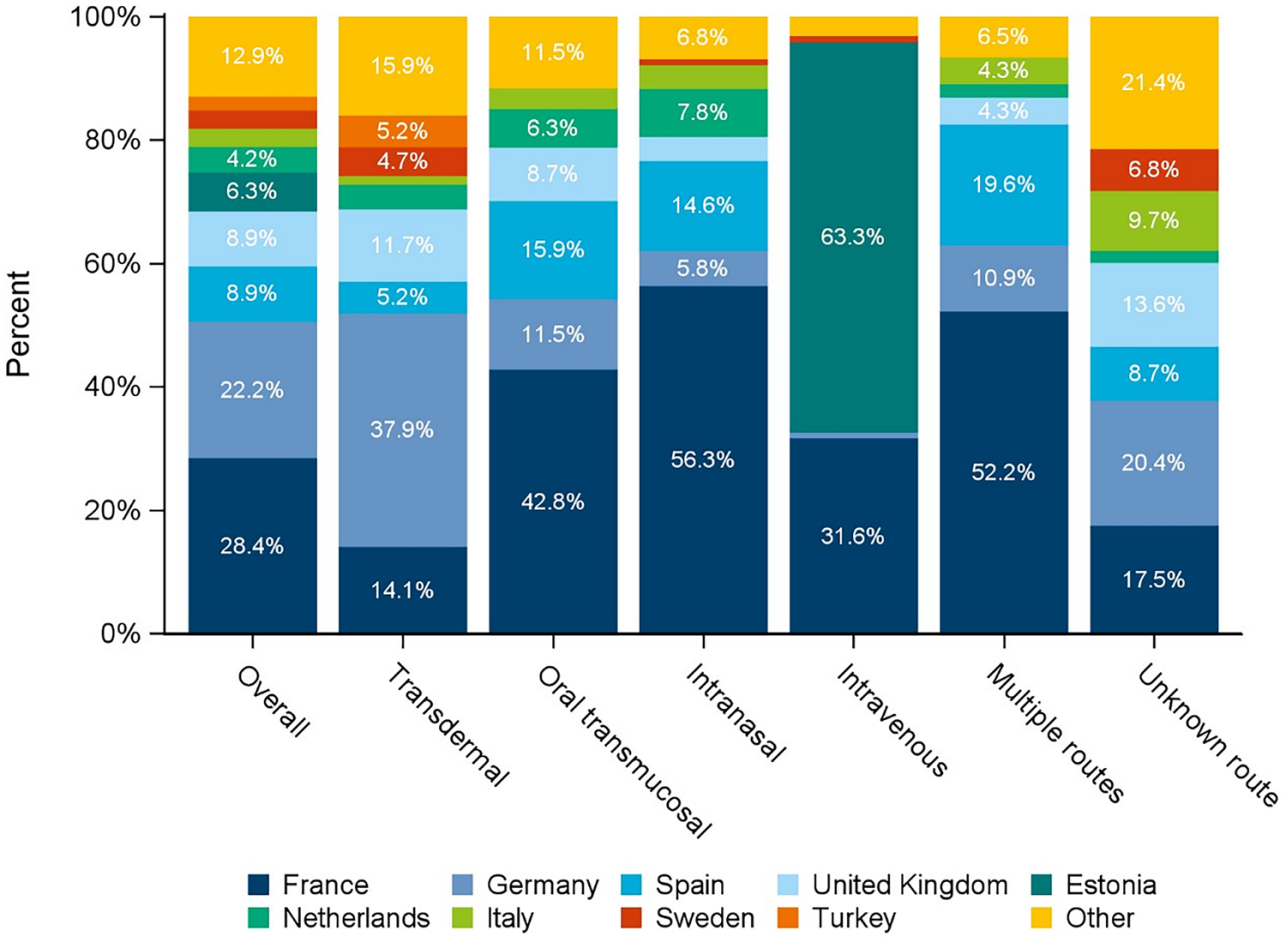
Reports of suspected fentanyl-associated abuse, dependence or withdrawal in Europe by (designated) route of administration and country (*N* = 985)

As displayed in Fig. 2, French and German reports constituted about half of all cases (28.4% and 22.2%, respectively). Reports referring to transdermal fentanyl were most often filed in Germany whereas TIRF products were mainly reported from France. Nearly two thirds of reports addressing intravenous fentanyl originated from Estonia.

Most reports were filed by physicians (47.1%), followed by pharmacists (21.3%, Table 1). The median age of cases was 45 years (*IQR*: 33–58) and 48.8% were female. Fentanyl had been used for a median duration of 65 days (*IQR*: 5–367). Among reports with a given indication or comorbidity (*N* = 858), 21.6% had musculoskeletal/connective tissue disorders and for 18.1%, neoplasms were reported. Depression was found in 10.7% of patients and 15.4% had a history of drug abuse, dependence or withdrawal. Common co-medications were benzodiazepines (18.6%) and antidepressants (12.4%) with other opioids reported in 25.0% of cases. Less than 5% of cases used drugs for opioid dependence. The use of recreational drugs was even less common. With respect to the case definition, reactions included in the sub-SMQ “drug abuse and dependence” were far more often coded than those referring to “drug withdrawal” (86.4% vs. 14.9%), see also Supplementary Fig. 1. Off label use was coded in 8.9% of cases whereas errors in prescribing and administration were coded for 2.6% and 7.7%, respectively. Nearly 40% of all cases had a prolonged hospital stay and 23.2% died.

**Table 1:** Characteristics of cases of suspected fentanyl-associated abuse, dependence or withdrawal in Europe overall and by potential indication

	**Overall (*N* = 985)**	**Musculoskeletal and connective tissue disorders**^**a**^** (*N* = 185)**	**Neoplasms benign, malignant and unspecified**^**d, a**^** (*N* = 155)**
**(Designated) route of administration**	***N* = 985**	***N* = 185**	***N* = 155**
Transdermal	43.4%	50.3%	29.7%
Oral transmucosal	21.1%	29.2%	31.0%
Intranasal	10.5%	9.2%	25.2%
Intravenous	10.0%	0.0%	0.6%
Multiple routes	4.7%	5.9%	11.0%
**Reporter**	***N* = 981**	***N* = 184**	***N* = 154**
Physician	47.1%	58.2%	59.7%
Pharmacist	21.3%	20.1%	16.2%
Other health professional	20.2%	10.3%	17.5%
Consumer/other non-health professional	11.4%	11.4%	6.5%
**Age (years)**	***N* = 546**	***N* = 137**	***N* = 107**
Median (IQR)	45 (33–58)	51 (41–65)	52 (42–65)
**Sex**	***N* = 926**	***N* = 182**	***N* = 150**
Female	48.8%	68.1%	49.3%
Male	51.2%	31.9%	50.7%
**Duration of fentanyl use (days)**	***N* = 193**	***N* = 35**	***N* = 33**
Median (IQR)	65 (5–367)	249 (8–731)	299 (91–393)
**Indication/comorbidity (history of…)**^**e**^	***N* = 858**	***N* = 185**	***N* = 155**
Musculoskeletal and connective tissue disorders^a^	21.6%	100%	21.3%
Neoplasms benign, malignant and unspecified^d, a^	18.1%	17.8%	100%
Depression (excl. suicide and self-injury)^b^	10.7%	24.9%	14.8%
Suicide, self-injury^b^	2.4%	2.7%	5.8%
Drug abuse, dependence or withdrawal^b^	15.4%	13.5%	8.4%
**Medication/drugs**^**e**^	***N* = 985**	***N* = 185**	***N* = 155**
Antidepressants	12.4%	25.9%	19.4%
Antipsychotics	3.4%	3.2%	4.5%
Benzodiazepines	18.6%	25.4%	20.0%
Other opioids	25.0%	37.8%	37.4%
Drugs for opioid dependence	4.6%	2.2%	5.8%
Alcohol	1.2%	0.5%	0.0%
Cannabis	1.8%	1.1%	2.6%
Cocaine	0.6%	1.1%	1.9%
**Selected reactions**^**e**^	***N* = 985**	***N* = 185**	***N* = 155**
Reactions referring to case definition			
Drug abuse and dependence^b^	86.4%	81.1%	92.9%
Drug withdrawal^b^	14.9%	22.2%	9.0%
Other reactions			
Off label uses^c^	8.9%	13.5%	9.0%
Product prescribing errors and issues^c^	2.6%	6.0%	2.6%
Product administration errors and issues^c^	7.7%	13.0%	6.5%
Accidental overdose^f^	1.3%	2.2%	0.7%
Suicide, self-injury^b^	5.7%	6.0%	6.5%
**Prolonged hospitalisation?**	***N* = 785**	***N* = 158**	***N* = 127**
Yes	39.5%	56.3%	40.9%
**Fatal outcome?**	***N* = 587**	***N* = 133**	***N* = 97**
Yes	23.2%	10.5%	17.5%

*IQR* interquartile range, For 33 cases, musculoskeletal/connective tissue disorders and neoplasms benign, malignant and unspecified were coded. Ns refer to the number of non-missing values for the respective characteristic

^a^System organ class (SOC)

^b^Standardised MedDRA Query (SMQ)

^c^high level term (HLT)

^d^incl. cysts and polyps

^e^multiple indications/comorbidities, medication/drugs, reactions possible

^f^preferred term (PT)

### Characteristics by route of administration

As displayed in Table 2 , among cases with a determined (designated) route of administration, the median age ranged from 31 (intravenous) to 50 years (oral transmucosal products) and the proportion of females from 34.7% (intravenous) to nearly 60% (TIRF). Fentanyl use was the shortest in cases with multiple routes (8 days) and longest for oral transmucosal products (366 days). Neoplasms were more often coded than musculoskeletal indications for intranasal products (40.2% vs. 17.5%) or multiple routes (38.5% vs. 25.6%) whereas the picture was reversed for transdermal fentanyl (12.6% vs. 25.4%) and (to a lesser extent) oral transmucosal products (26.8% vs. 30.2%). A history of drug abuse, dependence or withdrawal was most common in users of transdermal fentanyl and use of other opioids most often coded for TIRF. In terms of case definition, the proportion of reactions relating to drug abuse and dependence was the highest in cases using TIRF or multiple routes (> 97%) and drug withdrawal was most often recorded for intravenous (34.7%) and transdermal products (17.3%), see also Supplementary Fig. 1. With nearly 25%, off label use was most often coded for TIRF. Prescribing errors most often occurred with respect to oral transmucosal products and administration errors were most commonly recorded in users of transdermal and intranasal products, respectively. Prolonged hospital stays were most often found for intravenous fentanyl (66.7%) and fatal outcomes most common among users of transdermal products (35.2%). The 103 cases with an unknown route of administration were young (median 31 years) and often male (65.9%), a high proportion had a history of drug abuse, dependence or withdrawal (35.0%) and/or used benzodiazepines (34.0%) or other opioids (33.0%), and 42.9% died (data not shown).

**Table 2:** Characteristics of cases of suspected fentanyl-associated abuse, dependence or withdrawal in Europe by (designated) route of administration

	**Transdermal (** ***N*** ** = 427)**	**Oral transmucosal (** ***N*** ** = 208)**	**Intranasal (** ***N*** ** = 103)**	**Intravenous (** ***N*** ** = 98)**	**Multiple routes (** ***N*** ** = 46)**
**Reporter**	***N*** ** = 426**	***N*** ** = 207**	***N*** ** = 103**	***N*** ** = 98**	***N*** ** = 46**
Physician	52.8%	46.4%	49.5%	33.7%	41.3%
Pharmacist	17.4%	35.3%	37.9%	0.0%	30.4%
Other health professional	12.9%	14.0%	8.7%	64.3%	13.0%
Consumer/other non-health professional	16.9%	4.3%	3.9%	2.0%	15.2%
**Age (years)**	***N*** ** = 268**	***N*** ** = 103**	***N*** ** = 63**	***N*** ** = 3**	***N*** ** = 36**
Median (IQR)	44 (32–58.5)	50 (42–60)	47 (37–55)	31 (0–31)	49 (38.5–65)
**Sex**	***N*** ** = 394**	***N*** ** = 195**	***N*** ** = 103**	***N*** ** = 98**	***N*** ** = 45**
Female	47.0%	59.5%	59.2%	34.7%	55.6%
Male	53.0%	40.5%	40.8%	65.3%	44.4%
**Duration of fentanyl use (days)**	***N*** ** = 61**	***N*** ** = 42**	***N*** ** = 27**	***N*** ** = 31**	***N*** ** = 11**
Median (IQR)	10 (1–219)	366 (183–731)	299 (141–731)	8 (5–11)	342 (44–393)
**Indication/comorbidity (history of…)**^**e**^	***N*** ** = 366**	***N*** ** = 179**	***N*** ** = 97**	***N*** ** = 93**	***N*** ** = 43**
Musculoskeletal and connective tissue disorders^a^	25.4%	30.2%	17.5%	0.0%	25.6%
Neoplasms benign, malignant and unspecified^d, a^	12.6%	26.8%	40.2%	1.1%	39.5%
Depression (excl. suicide and self-injury)^b^	12.0%	11.2%	14.4%	1.1%	16.3%
Suicide, self-injury^b^	3.6%	1.7%	2.1%	0.0%	4.7%
Drug abuse, dependence or withdrawal^b^	20.8%	9.5%	7.2%	3.2%	2.3%
**Medication/drugs**^**e**^	***N*** ** = 427**	***N*** ** = 208**	***N*** ** = 103**	***N*** ** = 98**	***N*** ** = 46**
Antidepressants	13.3%	14.9%	15.5%	0.0%	19.6%
Antipsychotics	3.7%	3.4%	2.9%	0.0%	4.3%
Benzodiazepines	12.9%	15.9%	17.5%	32.7%	21.7%
Other opioids	23.7%	27.9%	28.2%	12.2%	26.1%
Drugs for opioid dependence	4.0%	2.9%	4.9%	0.0%	2.2%
Alcohol	1.4%	0.0%	1.0%	0.0%	0.0%
Cannabis	1.4%	1.4%	1.0%	0.0%	4.3%
Cocaine	0.5%	0.5%	0.0%	0.0%	2.2%
**Selected reactions**^**e**^	***N*** ** = 427**	***N*** ** = 208**	***N*** ** = 103**	***N*** ** = 98**	***N*** ** = 46**
Reactions referring to case definition					
Drug abuse and dependence^b^	84.8%	97.1%	99.0%	65.3%	97.8%
Drug withdrawal^b^	17.3%	3.4%	2.9%	34.7%	4.3%
Other reactions					
Off label uses^c^	1.4%	24.5%	24.3%	0.0%	8.7%
Product prescribing errors and issues^c^	0.5%	10.1%	1.9%	0.0%	2.2%
Product administration errors and issues^c^	12.2%	2.4%	9.7%	0.0%	4.3%
Accidental overdose^f^	1.6%	0.5%	1.0%	0.0%	0.0%
Suicide, self-injury^b^	8.7%	3.4%	2.9%	0.0%	2.2%
**Prolonged hospitalisation?**	***N*** ** = 376**	***N*** ** = 167**	***N*** ** = 85**	***N*** ** = 36**	***N*** ** = 43**
Yes	37.5%	34.1%	35.3%	66.7%	48.8%
**Fatal outcome?**	***N*** ** = 273**	***N*** ** = 117**	***N*** ** = 67**	***N*** ** = 35**	***N*** ** = 32**
Yes	35.2%	4.3%	9.0%	2.9%	3.1%

*IQR *interquartile range, For 103 cases, no designated route of administration could be established. Ns refer to the number of non-missing values for the respective characteristic

^a^System organ class (SOC)

^b^Standardised MedDRA Query (SMQ)

^c^high level term (HLT)

^d^incl. cysts and polyps

^e^multiple indications/comorbidities, medication/drugs, reactions possible

^f^preferred term (PT)

### Characteristics by outcome

The highest numbers of fatal outcomes were observed between 2015 and 2017 (16–19 per year, Supplementary Fig. 2). Cases with a fatal outcome were younger (median 33 vs. 48 years), more often male than those surviving the adverse reaction (67.2% vs. 46.5%); they also suffered less often from musculoskeletal disorders or neoplasms but frequently had a history of drug abuse, dependence or withdrawal (35.5% vs. 14.4%, Supplementary Table 2). Nearly 53% of fatal reports were filed by other health professionals and consumers/other non-health professionals as compared to 16.3% of non-fatal reports. An accidental overdose was coded in 8.8% of fatal cases. The most common causes of death (available for 89 fatal cases) were assigned to injury, poisoning and procedural complications with toxicity to various agents coded in 30.3%.

Users of transdermal fentanyl were treated with a median dose of 75 μg per hour (Supplementary Table 3). In 61.5% of fatal and 83.1% of non-fatal cases (overall 73.1%), there was no evidence of another route of administration other than the transdermal. The most common route in fatal cases other than the intended route was an intravenous administration, whereas among non-fatal cases, oral use was most common.

### Characteristics by potential indication

The median age of patients with musculoskeletal/connective tissue disorders was 51 years and 68.1% were female, whereas those with neoplasms (median age: 52 years) showed an equal sex ratio (Table 1). A diagnosis of depression was more likely in cases with musculoskeletal/connective tissue disorders than in those with neoplasms (24.9% vs. 14.8%) as was a history of drug abuse, dependence or withdrawal (13.5% vs. 8.4%), a comedication of benzodiazepines (25.4% vs. 20.0%) or a reaction related to drug withdrawal (22.2% vs. 9.0%). In contrast, patients with musculoskeletal/connective tissue disorders less often used drugs for opioid dependence (2.2% vs. 5.8%) or had a coded reaction indicating drug abuse or dependence (81.1 vs. 92.9%).

For both indications, reports were more often filed by physicians, the duration of fentanyl use was considerably longer and the use of antidepressants, benzodiazepines or other opioids more common than in all other cases.

A prolonged hospitalisation was more common among cases with musculoskeletal/connective tissue disorders (56.3%) than in those with neoplasms (40.9%), whereas the picture was reversed for fatal outcomes (10.5% vs. 17.5%).

## Discussion

This study included almost 1000 reports of fentanyl abuse, dependence or withdrawal from Europe. Of those nearly 90% were linked to a (designated) route of administration. Case characteristics varied substantially by route in terms of indication, abuse history, coded reactions and fatality. There was an unexpected high proportion of cases with depression or a prolonged hospitalisation especially in those with musculoskeletal disorders indicating complex treatment situations and high economic impact.

### Transdermal fentanyl

More than 40% of the reports referred to fentanyl patches which is in accordance with their comparatively broad indication [3].

The high share of fatalities in this group seems in line with post-mortem analyses from Germany [18]. However, several factors indicate that the cases in our study, at least in part, do not reflect typical users of transdermal fentanyl. They were younger and more likely to be male when compared to overall users of transdermal fentanyl in Germany [19] and the median dose of 75 μg per hour exceeds the maximum of daily morphine equivalents recommended for non-cancer pain [20, 21]. Furthermore, a history of drug abuse and dependence was coded in 20% of cases. The short duration of use and a comparatively high reporting frequency by consumers or other non-health professionals indicate that use did not always originate from appropriate pain therapy.

For more than 25% of all and nearly 40% of the fatal cases, a route other than the intended transdermal application was coded. Transdermal fentanyl can be misused for example by extracting the drug into a liquid and injecting it or sucking/swallowing pieces of a cut patch [22]. A disproportionally higher involvement of intravenous rather than oral misuse was observed in fatal outcomes. This seems plausible since oral intake is subject to first-pass metabolism [2]. Accordingly, an analysis of acute health events recorded by poison centres found that death or major effects occurred more often with intentional prescription opioid abuse via non-oral routes of administration than ingestion alone [23]. Although newer patch formulations contain lower amounts of drug, a substantial amount of fentanyl remains in a patch after 3 days of use. Therefore, discarded patches, e.g. in hospital garbage, can attract addicts. Educating patients and care givers regarding safe disposal of fentanyl patches can thus improve the drug’s overall safety [24]. Furthermore, the development of tamper-resistant patches might hinder their non-medical use [25].

### Transmucosal immediate-release fentanyl

Since all TIRF products share the same indication with minor differences in terms of pharmacokinetics and dosing intervals [1], it was not surprising that the respective cases were similar in most characteristics. We, however, observed differences with respect to indication and coded reactions. In our analysis, about 60% of intranasal users and nearly 75% of users of transmucosal fentanyl had no cancer diagnosis and more than 70% in both groups used no other (maintenance) opioids. Although comparisons are hampered by the different data, these proportions are substantially higher than in other European studies addressing the overall use of oral transmucosal and intranasal fentanyl. In a post-marketing study from England, for instance, about 38% of patients treated with buccal fentanyl had no breakthrough cancer pain and only 30% had no regular opioid prescribed [26]. A Spanish analysis found that patients without previous cancer or breakthrough cancer pain represented 27% of new users of immediate-release fentanyl [5]. In this respect, problematic prescribing was reported from German physicians attending pain conferences where 90% had knowledge of non-indicated prescriptions of rapid-onset preparations of fentanyl or were involved in these [6].

Finally, the high proportion of physicians and pharmacists reporting abuse, dependence or withdrawal in TIRF indicates treatment mainly within the health-care system. The fact that more than one third of the reports were issued by pharmacists might hint at abuse or dependence, especially when prescribing was done by several physicians (“doctor shopping”), which was observed in a French study [27].

### Fentanyl use in musculoskeletal disorders, comorbidity and outcome

One of the most striking results of our analysis was the high percentage of patients with depression or prolonged hospitalisations, respectively. These proportions were especially high in those with musculoskeletal disorders where one in four cases was diagnosed with depression and more than half had a prolonged hospital stay. Together with the comparatively long duration of fentanyl use and the large number of patients also using benzodiazepines, these findings are concerning. An association between depression and chronic pain and/or long-term opioid use is known [28–30], and depression has been found to increase the clinical and economic impact of chronic pain conditions, e.g. in terms of sick days [31] and total health costs [32].

Furthermore, studies have shown an involvement of depression and benzodiazepines in abuse/misuse-related hospitalisations [33, 34]. Compared with Schifano et al. who also examined EudraVigilance data, but with a shorter observation period and no restriction to European countries, benzodiazepine use was more common in our study, whereas other opioids but also recreational drugs such as alcohol or cannabis were coded far less often [13]. Interestingly, reactions indicating a recreational fentanyl use were not recorded at all in our study, although an underreporting of these drugs or habits seems likely.

Opioid withdrawal might also occur when opioids are taken as prescribed under medical supervision, and withdrawal is considered the main barrier to opioid discontinuation [35]. The long duration of use and the comparatively large proportion of coded reactions referring to drug withdrawal in cases with musculoskeletal disorders might therefore indicate (unguided) attempts to discontinue/taper fentanyl treatment. This has been reported to be especially challenging for transdermal systems (for which in terms of route the highest percentage of withdrawal was reported). Besides individualised strategies and a reasonable timeline, a conversion to oral opioids might be necessary at least at later stages of the process [36]. If such tapering attempts were associated with (prolonged) hospitalisations, this could not be determined from the data.

Another important finding of our study was the high mortality with nearly one quarter of cases having a fatal outcome. However, Schifano et al. found similar proportions of prolonged hospitalisations but a higher percentage (one third) of fatal outcomes [13]. Although a comparison is hampered by different case definitions, one might conclude that fatality of reported cases of fentanyl abuse, dependence or withdrawal is lower in Europe compared to other regions of the world.

The high percentage of intravenous fentanyl use in 2017 might explain the maximum of fatal cases we observed that year. Furthermore, increases in the quantities seized of, among others, fentanyl derivatives have been reported in Europe around this time period [12] which probably added to these findings. In our study, cases with fatal outcomes were mainly characterised by no coded indications, short durations of use and a history of drug abuse, dependence or withdrawal. Interestingly, other drugs (except those used for opioid dependence and recreational drugs) were used less often than in non-fatal cases, and toxicity to various agents was coded in only 30% of fatal cases. This might point at fentanyl as the sole cause of death in a substantial percentage of cases. However, the qualifications of the persons reporting fatal and non-fatal cases varied substantially indicating different reporting situations. Accordingly, the lower proportion of reported co-medication in fatal cases might be attributed to a more limited access to the respective information for other health professionals and consumers/non-health professionals as compared to physicians and pharmacists. Given that for only one in eight fatal cases suicide/self-injury was coded, and an accidental overdose in every 12th fatal case, one can assume that most respective events were not intentional.

Finally, transmucosal or transdermal formulations of fentanyl are convenient treatment options providing pain relief immediately or over an extended period, respectively. However, the risks for adverse events including fatalities, even when used as prescribed, are not to be underestimated. Critical circumstances in this regard include treatment initiation in opioid-naïve patients, too high (initial) doses or the involvement of other drugs affecting the central nervous system [19, 37]. These risks should be kept in mind as should the biological, psychological and social components of pain [38] when considering initiation or continuation with these medications.

### Strengths and limitations

The major strength of this study is the large series of reports from multiple different countries and that most of them could be linked to a (designated) route of administration. This study, however, is subject to several limitations attributed to the nature of the underlying data. Spontaneous reporting systems suffer from underreporting [39] which is disproportionally often observed for older drugs and known adverse events [40]. Therefore, since abuse, dependence and withdrawal are typical adverse events for all opioids and fentanyl is a well-established drug, underreporting is likely and may be more prominent for the older transdermal formulations than for TIRF products.

Whether fentanyl was prescribed by a physician or obtained via illegal sources or if fentanyl derivatives were coded as fentanyl cannot be determined by the data, however, the large proportion of physicians and pharmacists reporting at least for the transdermal route and TIRF products indicates that fentanyl treatment occurred in the usual medical care settings.

Another limitation of this type of data is the quality and completeness of the provided information. Some variables such as the dose or duration of use were only scarcely filled; furthermore, the actual route of administration might be underreported. The same might apply to the potential indication hampering further analyses. However, we included as much information as possible using several free texts.

The SMQ “drug abuse, dependence and withdrawal” includes aberrant behaviours subsumed as opioid use disorder but also dependence and withdrawal symptoms. Consequently, our case definition yielded a heterogenous group of fentanyl (mis)users which might differ from classification or diagnosing systems such as the Diagnostic and Statistical Manual of Mental Disorders (DSM) or the International Classification of Diseases (ICD). Furthermore, reporting differences by reporters’ qualifications or countries and changes over time might have influenced our findings. However, the aim of this study was a broad insight into fentanyl-associated abuse, dependence or withdrawal reported from Europe.

Finally, given the reporting limitations and since the number of fentanyl users neither overall nor by (designated) route of administration is not known, it is not possible to determine any risks of abuse, dependence or withdrawal in users of respective preparations.

## Conclusions

Our study found that in suspected cases of abuse, dependence or withdrawal, fentanyl was mainly used for non-cancer indications and most often as transdermal formulations. Half of the patients with musculoskeletal disorders used transdermal fentanyl and depression as well as prolonged hospitalisations were most common in this group. This phenomenon of (often) long-term fentanyl users, many of them presumably in regular treatment, might have a high impact on morbidity, mortality and the healthcare system.

## Supplementary Information

Below is the link to the electronic supplementary material.

Supplementary file1 (DOCX 182 KB)

## Data Availability

The data that support the findings of this study are available from the European Medicines Agency (EMA) but restrictions apply to the availability of these data, which were used under license for the current study, and so are not publicly available. Data are, however, available from the authors upon reasonable request and with permission of EMA.
