# Supplementary material for: Abuse, dependence and withdrawal associated with fentanyl and the role of its (designated) route of administration: an analysis of spontaneous reports from Europe
Source: Eur J Clin Pharmacol. 2022 Dec 16;79(2):257–67. doi: 10.1007/s00228-022-03431-x (PMC9879804; doi:10.1007/s00228-022-03431-x)
Supplement: Supplementary file 1 — Supplementary file1 (DOCX 182 KB) [file 228_2022_3431_MOESM1_ESM.docx]

**Supplement**

**Supplementary Table 1** Reactions (preferred terms) referring to the SMQ “Drug abuse, dependence and withdrawal” with its sub-SMQs “Drug abuse and dependence” and “Drug withdrawal”

| **Drug abuse and dependence** | **Drug withdrawal** |
| --- | --- |
| Dopamine dysregulation syndrome | Drug withdrawal convulsions |
| Drug abuse | Drug withdrawal headache |
| Drug abuser | Drug withdrawal maintenance therapy |
| Drug dependence | Drug withdrawal syndrome |
| Drug dependence, antepartum | Drug withdrawal syndrome neonatal |
| Drug dependence, postpartum |  |
| Drug use disorder |  |
| Drug use disorder, antepartum |  |
| Drug use disorder, postpartum |  |
| Intentional overdose |  |
| Intentional product misuse |  |
| Maternal use of illicit drugs |  |
| Neonatal complications of substance abuse |  |
| Substance abuse |  |
| Substance abuser |  |
| Substance dependence |  |
| Substance use disorder |  |

SMQ: Standardised MedDRA Query

**Supplementary Table 2** Characteristics of cases of suspected fentanyl-associated abuse, dependence or withdrawal in Europe by outcome

|  | **Not fatal (N=451)** | **Fatal  (N=136)** |
| --- | --- | --- |
| **Reporter** | **N=449** | **N=135** |
| Physician | 60.6% | 43.0% |
| Pharmacist | 23.2% | 4.4% |
| Other health professional | 9.4% | 26.7% |
| Consumer/other non-health professional | 6.9% | 25.9% |
| **Age (years)** | **N=290** | **N=87** |
| Median (IQR) | 48 (36-61) | 33 (26-46) |
| **Sex** | **N=445** | **N=122** |
| Female | 53.5% | 32.8% |
| Male | 46.5% | 67.2% |
| **Duration of fentanyl use (days)** | **N=153** | **N=11** |
| Median (IQR) | 87 (6-393) | 2 (1-183) |
| **Indication/comorbidity (history of…)*** | **N=411** | **N=110** |
| Musculoskeletal and connective tissue disorders^†^ | 29.0% | 12.7% |
| Neoplasms benign, malignant and unspecified^¥,†^ | 19.5% | 15.5% |
| Depression (excl. suicide and self-injury)^§^ | 15.1% | 9.1% |
| Suicide, self-injury^§^ | 2.9% | 5.5% |
| Drug abuse, dependence or withdrawal^§^ | 14.4% | 35.5% |
| **Medication/drugs*** | **N=451** | **N=136** |
| Antidepressants | 17.3% | 11.8% |
| Antipsychotics | 5.1% | 2.2% |
| Benzodiazepines | 23.7% | 17.6% |
| Other opioids | 30.4% | 20.6% |
| Drugs for opioid dependence | 3.1% | 8.8% |
| Alcohol | 0.9% | 3.7% |
| Cannabis | 0.2% | 5.1% |
| Cocaine | 0.0% | 1.5% |
| **Designated route** | **N=451** | **N=136** |
| Transdermal | 39.2% | 70.6% |
| Oral transmucosal | 24.8% | 3.7% |
| Intranasal | 13.5% | 4.4% |
| Intravenous | 7.5% | 0.7% |
| Multiple routes | 6.9% | 0.7% |
| **Selected reactions*** | **N=451** | **N=136** |
| Reactions referring to case definition |  |  |
| Drug abuse and dependence^§^ | 78.3% | 98.5% |
| Drug withdrawal^§^ | 23.3% | 1.5% |
| Other reactions |  |  |
| Off label uses**^‡^** | 12.4% | 2.2% |
| Product prescribing errors and issues**^‡^** | 3.1% | 0.0% |
| Product administration errors and issues**^‡^** | 5.3% | 19.1% |
| Accidental overdose^¶^ | 0.0% | 8.8% |
| Suicide, self-injury^§^ | 5.1% | 11.8% |
| **Commonly reported causes of death** |  | **N=89** |
| Injury, poisoning and procedural complications**^†^** |  | 60.7% |
| Toxicity to various agents^¶^ |  | 30.3% |
| Psychiatric disorders**^†^** |  | 31.5% |
| Respiratory, thoracic and mediastinal disorders**^†^** |  | 22.5% |

^†^System organ class (SOC), ^§^Standardised MedDRA Query (SMQ), ^‡^High level term (HLT), ), ^¶^Preferred term (PT), ^¥^ incl. cysts and polyps,

*Multiple indications/comorbidities, medication/drugs, reactions possible

IQR: interquartile range

Ns refer to the number of non-missing values for the respective characteristic.

**Supplementary Table 3** Characteristics of cases of suspected abuse, dependence or withdrawal associated with transdermal fentanyl in Europe by outcome

|  | **Not fatal  (N=177)** | **Fatal (N=96)** |
| --- | --- | --- |
| **Indication/comorbidity (history of…)** | **N=163** | **N=77** |
| Drug abuse, dependence or withdrawal^§^ | 19.6% | 35.1% |
| **Duration of fentanyl use (days)** | **N=44** | **N=5** |
| Median (IQR) | 35.5 (2-265.5) | 1 (1-2) |
| **Dose per hour (microgram)** | **N=85** | **N=23** |
| Median (IQR) | 75 (50-100) | 75 (75-100) |
| **Actual route of administration** | **N=177** | **N=96** |
| No evidence of other than transdermal route | 83.1% | 61.5% |
| Evidence of another actual route of administration | **N=30** | **N=37** |
| Oral (includes chewing or buccal/sublingual application) | 56.7% | 27.0% |
| Nasal/inhalation (includes smoking) | 23.3% | 18.9% |
| Intravenous | 6.7% | 40.5% |
| Multiple routes | 13.3% | 13.5% |

^§^Standardised MedDRA Query (SMQ)

IQR: interquartile range

Ns refer to the number of non-missing values for the respective characteristic.


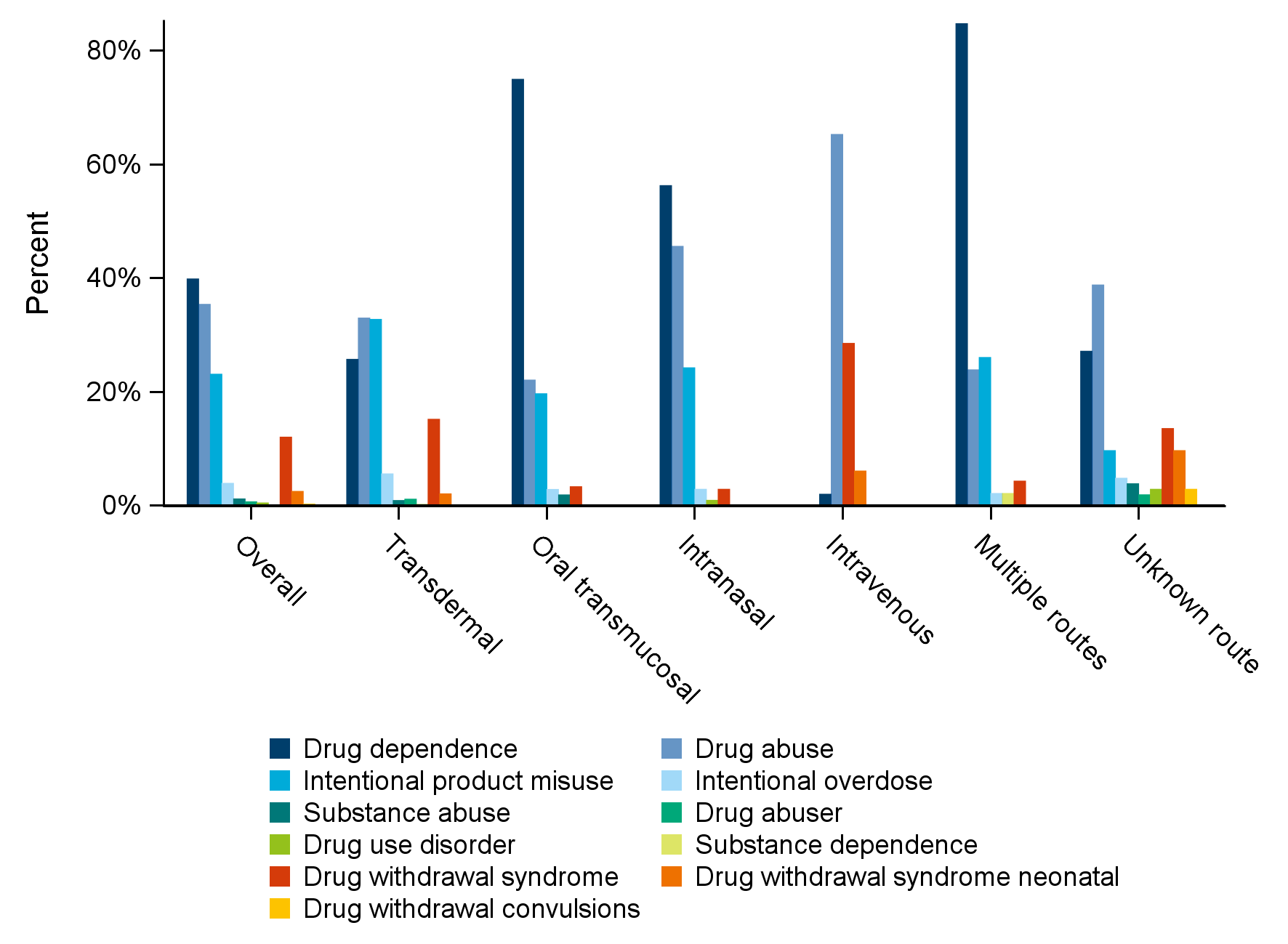


**Supplementary Fig 1.** Reactions (preferred terms) referring to the SMQ “Drug abuse, dependence and withdrawal” by route of administration

SMQ: Standardised MedDRA Query
Multiple reactions per case were possible.


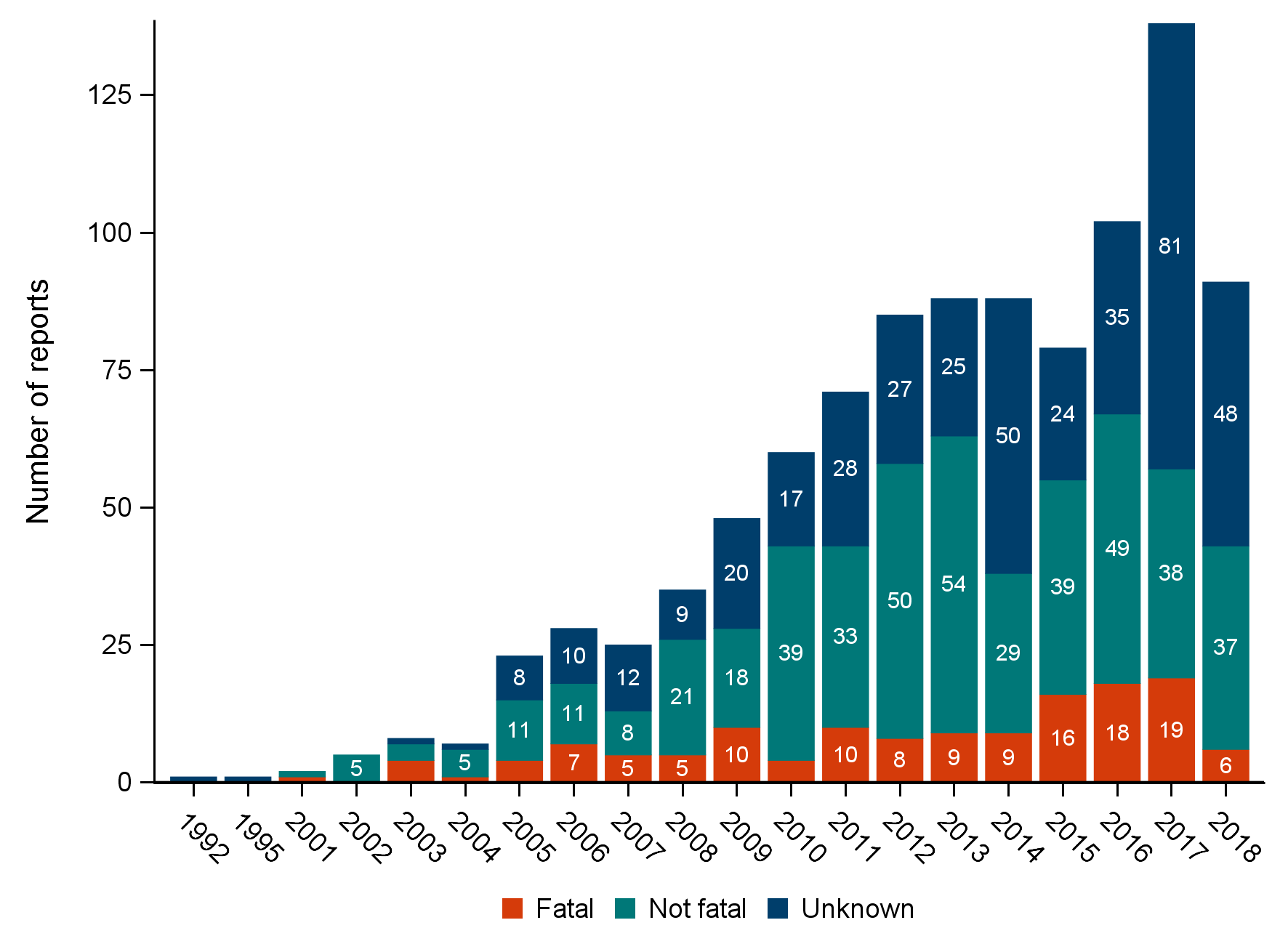


**Supplementary Fig 2.** Reports of suspected fentanyl-associated abuse, dependence or withdrawal in Europe by outcome and year
